# Peptide microarrays coupled to machine learning reveal individual epitopes from human antibody responses with neutralizing capabilities against SARS-CoV-2

**DOI:** 10.1080/22221751.2022.2057874

**Published:** 2022-04-11

**Authors:** Sven-Kevin Hotop, Susanne Reimering, Aditya Shekhar, Ehsaneddin Asgari, Ulrike Beutling, Christine Dahlke, Anahita Fathi, Fawad Khan, Marc Lütgehetmann, Rico Ballmann, Andreas Gerstner, Werner Tegge, Luka Cicin-Sain, Ursula Bilitewski, Alice C. McHardy, Mark Brönstrup

**Affiliations:** a Helmholtz Centre for Infection Research, Braunschweig, Germany; b Braunschweig Integrated Centre of Systems Biology (BRICS), Technische Universität Braunschweig, Braunschweig, Germany; c Partner Site Hannover-Braunschweig, German Centre for Infection Research (DZIF), Germany; d University Medical Centre Hamburg-Eppendorf, Hamburg, Germany; e Bernhard Nocht Institute for Tropical Medicine, Hamburg, Germany; f Partner Site Hamburg-Lübeck-Borstel-Riems, German Centre for Infection Research, Germany; g Center for Diagnostics, Institute of Medical Microbiology, Virology and Hygiene, University Medical Center Hamburg-Eppendorf (UKE), Hamburg, Germany; h Institut für Biochemie, Biotechnologie du Bioinformatik, Abteilung Biotechnologie, Technische Universität Braunschweig, Braunschweig, Germany; i Klinikum Braunschweig, Hals-, Nasen-, Ohrenklinik, Braunschweig, Germany; j Biomolecular Drug Research Center (BMWZ), Hannover, Germany

**Keywords:** COVID-19, SARS CoV-2, immunoassays, peptide arrays, serology, machine learning, neutralizing antibodies

## Abstract

The coronavirus SARS-CoV-2 is the causative agent for the disease COVID-19. To capture the IgA, IgG, and IgM antibody response of patients infected with SARS-CoV-2 at individual epitope resolution, we constructed planar microarrays of 648 overlapping peptides that cover the four major structural proteins S(pike), N(ucleocapsid), M(embrane), and E(nvelope). The arrays were incubated with sera of 67 SARS-CoV-2 positive and 22 negative control samples. Specific responses to SARS-CoV-2 were detectable, and nine peptides were associated with a more severe course of the disease. A random forest model disclosed that antibody binding to 21 peptides, mostly localized in the S protein, was associated with higher neutralization values in cellular anti-SARS-CoV-2 assays. For antibodies addressing the N-terminus of M, or peptides close to the fusion region of S, protective effects were proven by antibody depletion and neutralization assays. The study pinpoints unusual viral binding epitopes that might be suited as vaccine candidates**.**

## Introduction

Coronaviruses have become a major threat for human health since the occurrence of three global outbreaks in the past two decades, caused by SARS-CoV-1 in 2003, MERS-CoV in 2012, and SARS-CoV-2 in 2019. The latter is the causal agent of COVID-19, a disease that has infected more than 430 million people worldwide and caused more than 5.9 million deaths (as of 24 February 2022). In addition, human coronaviruses (OC43, NL63, 229E, and HKU1) exist that are widespread and cause mainly mild or flu-like disease symptoms. To develop effective treatments that take the stage and severity of the disease into account, it is of prime importance to gain an understanding of the cellular and humoral immune response against SARS-CoV-2.

Antibody formation can be detected on a routine, large-scale basis by specific enzyme-linked immunosorbent assays (ELISA) or chemiluminescent immune assays (CLIA) [1,2]. Such assays indicate whether binding antibodies directed against one of the major structural proteins of SARS-CoV-2 – usually the receptor-binding domain (RBD), other regions of the spike protein or the nucleocapsid protein – exist, but these assays cannot identify the individual epitopes on the viral proteins targeted by polyclonal sera. Such privileged, immunogenic epitopes would be prime candidates for precision diagnostics, vaccination approaches, or therapeutic antibodies, in particular, if their recognition is associated with neutralization. To characterize antibodies at the epitope level, microarrays of short synthetic peptides as antibody baits have been successfully applied for a variety of disease conditions, including viral infections [3–5]. Therefore, microarrays have been quickly adapted to investigate SARS-CoV-2 infections. First reports studied 29 and 10 patients from China [6,7] and 43 patients from Japan [8] with respect to IgM and IgG responses, and longitudinal responses with three patients from Germany [9]. Other studies particularly focused on the differentiation of a SARS-CoV-2 response against other human coronaviruses using soluble DNA-barcoded peptide libraries [10] and microarrays [11,12]. To identify epitopes that elicit neutralizing antibodies, Poh et al. used soluble peptides spanning the spike protein, and Li et al. enriched such antibodies using peptides identified from microarray experiments [13,14]. Heffron et al. used ultradense microarrays that covered the full proteome of SARS-CoV-2 and eight other coronaviruses to characterize the IgG response in 40 patients and 20 controls [15]. Epitope signatures of patients with graded disease severity were described by Schwarz et al. [16].

In this study, we aimed to define the humoral immune response against SARS-CoV-2 at an individual epitope resolution in 67 positive and 22 negative samples. We were interested in characterizing the immunodominant epitope repertoire, applied machine learning methods to ascribe the SARS-CoV-2 neutralizing capabilities of the polyclonal sera to individual epitope contributions, and verified the functional relevance of two prominent epitopes experimentally. Thus, the study disclosed known as well as hitherto unknown epitopes that might be valuable targets for immunotherapeutics.

## Materials and methods

### Sample information

In total, 67 heat-inactivated serum samples were obtained from 36 infected patients who were positively diagnosed by RT-PCR. These patients were admitted to two German hospitals (Klinikum Braunschweig and University Medical Center Hamburg–Eppendorf, Hamburg) between March and June 2020. The majority of these patients experienced a mild course of the disease. For 17 patients, two or more sera were available for a longitudinal monitoring of their immune responses. Twenty-two SARS-CoV-2 negative patient sera were obtained from different sources. Six pre-pandemic sera were collected as a control group in a MERS vaccine study before the outbreak of SARS-CoV-2 in 2019. Thirteen samples were collected from patients during the first outbreak of SARS-CoV-2 in Germany (Q1/2020) but diagnosed to be negative for the virus via RT–PCR. Three pre-pandemic serum samples were obtained from healthy volunteers at HZI from 2011 to 2016. The information on patient samples, including age, sex, disease severity, or weeks post-infection at the time of sampling is summarized in Supplemental Data File 1.

Donors provided written informed consent. The protocol was approved by the Ethics Committee of the Hamburg Medical Association, Germany (PV7298), and by the Commission for Ethical Issues of the TU Braunschweig (FV-2020-02), for the samples from Hamburg and Braunschweig, respectively.

### Peptide synthesis and slide preparation

**Figure 1.**
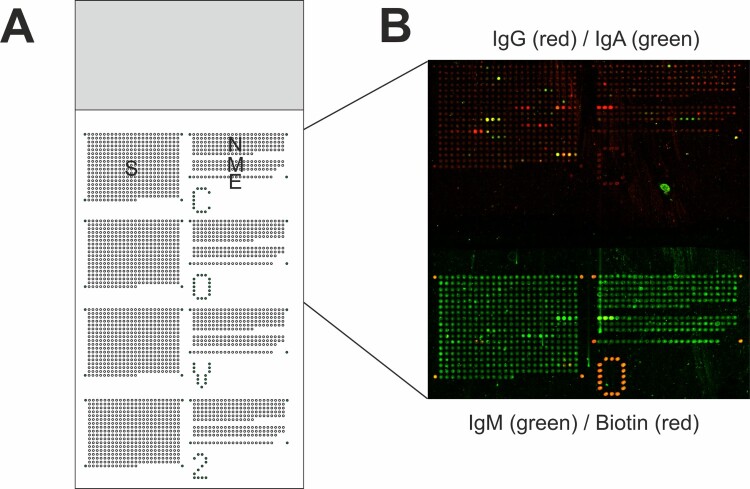
Slide layout and exemplary data of fluorescent signals obtained for sample ID f. (A) Each slide holds 2592 peptide sequences and 86 biotin control spots arranged in four sub-grids. Each sub-grid contains 648 15mer peptides with an offset of three amino acids to the next peptide, encompassing the whole sequences of S, N M, and E protein derived from ref. seq. NC_045512. (B) Bound antibodies against SARS-CoV-2 peptide sequences were visualized with isotype-specific anti-human secondary antibodies coupled to different fluorescent dyes or fluorescently labelled streptavidin. In one sub-grid, IgG (red) and IgA (green) antibodies were detected. Antibodies bound to the same peptide sequences produce yellow signals as overlay of red and green signals. In the adjacent sub-grid, IgM (green) and biotin (red) were detected.

Peptides were synthesized via Fmoc solid-phase peptide synthesis in an Intavis peptide synthesizer (Tübingen, Germany) on cellulose membranes by the SPOT method as described earlier [3,17,18]. In total, 648 peptides with a length of 15 amino acids and an offset of three amino acids to the following peptide of the protein sequences S, N, M and E derived from Ref. NC_045512 (Wuhan-Hu-1) were produced. All peptide sequences are given in Supplemental Data File 1. After processing, cellulose-peptide conjugates were spotted onto glass slides with an Affymetrix 428 Ring-Pin Spotter (CA, USA) to obtain peptide microarrays with four identical sub-grids. In addition, 86 cellulose-biotin-conjugates were printed as controls onto each glass slide. The procedure ensures that equal amounts of biochemically different peptides are spotted onto the glass surface using cellulose fibres as linkers [18]. The exact layout is shown in Figure 1.

### Incubation of peptide microarrays

Peptide microarrays were incubated as follows: Arrays were washed with 100% EtOH for three min, and three times with tris-buffered saline (TBS; 50 mM Tris, 150 mM NaCl, 30 mM KCl, pH 7.0) for 3 min each. To reduce unspecific binding, slides were blocked overnight in a blocking buffer that consisted of 2% casein in TTBS (1% Tween 20 (w/v) in TBS). Due to the sufficient signal–noise ratios, a pre-incubation of samples with cellulose/linker matrix as reported for other sample matrices [3], was not necessary and therefore not performed. Antibody-containing crude serum samples were first heat inactivated by 56°C for 30 min in order to eliminate their infectivity [19]. Then, the sera were diluted 1:120 in blocking buffer. Slides were equipped with a sealing system (Grace Bio-Labs HybriWell, GBL612106, 100 µl; OR, USA) spanning two sub-grids. Diluted primary antibody-containing samples were carefully deposited in each chamber and incubated at 4°C overnight. Thereafter, slides were washed three times with TTBS for 5 min per wash. Bound antibodies were stained using isotype-specific anti-human antibodies conjugated to fluorescent dyes. Secondary antibodies were diluted in LowCrossBuffer^®^ Strong (Candor Bioscience, Germany) as follows: Alexa Fluor® 647 anti-human IgG 1:960, Cy™3 anti-human IgA 1:720, Cy™3 anti-human IgM 1:4800, and Cy^TM^5-Streptavidin 1:240 (109-606-033, 109-166-01, 109-166-129 and 016-170-084, Jackson ImmunoResearch, PA, USA). For differential staining, slides were equipped with one sub-grid encompassing chamber (GBL612107, 50 µl; OR, USA) prior to incubation for 90 min at room temperature in the dark. Unbound secondary antibodies were removed via washing slides two times with TTBS and three times with ddH_2_O. Immediately thereafter, slides were dried in a stream of nitrogen and fluorescent readouts were obtained using an Agilent DNA Microarray Scanner (CA, USA). Fluorescent read files were processed using the Agilent Feature Extraction Software (12.1.1.1). The high variance in the signal-to-noise ratios across all samples did not allow the use of an automated SPOT calling procedure. Therefore, data mining was done by visual inspection of 16-bit tiff files. Two experienced scientists classified signals as positive or negative independently. Peptides reacting positively were marked for all three secondary antibodies used. Secondary antibodies were tested for unspecific binding to synthesized peptides prior to the investigation of serum samples and the binding signals were excluded from the data set. True positive signals were called if signals appeared in both individual reads. The monoclonal IgG antibody SH2029-G6 was obtained from the Institut für Biochemie, Biotechnologie und Bioinformatik (TU Braunschweig, Germany). Mapping of antibodies to 3D structures was done using Chimera 1.13.1 on Darwin64 [20].

### Antibody depletion assay

The depletion of specific antibodies from sera was achieved via their corresponding antigen peptides that were bound to sepharose beads. Sera were incubated with the peptide-bound beads and subsequently eluted. The flow through should be depleted from antibodies binding to the peptides. In brief, the peptide MADSNGTITVEELKKC corresponding to sequence M1 was synthesized via Fmoc solid-phase peptide synthesis with the addition of a C-terminal cysteine residue for coupling. The peptide PPLLTDEMIAQYTSAC, corresponding to S288, was obtained from GeneCust (Boynes, France). As a control peptide for a mock treatment, the sequence CMGVADLIKKFESISKEE was used. For sepharose coupling, 5 mg bovine serum albumin (BSA) in 200 µl phosphate-buffered saline (PBS) was incubated with 1 mg of Sulfo-M-maleimidobenzoyl-N-hydroxysuccinimide ester (Sulfo-MBS; ThermoFisher Scientific, MA, USA) in PBS for 30 min at room temperature. Thereafter 50 µl of the crosslinked BSA-MBS was incubated with 166 ng peptide solved in 10 µl DMSO on an end-over-end shaker for 3 h at room temperature. 200 mg of CNBr activated sepharose beads (Cytiva, WA, USA) were swollen with 1 ml HCl (1 mM) for 2 h and washed with 50 ml HCl (1 mM) using a vacuum flask equipped with a 3G glass frit. The beads were immediately washed with coupling buffer (0.1 M NaHCO_3_; 0.5 M NaCl; pH 8.3) and 40 mg thereof was transferred to 500 µl microspin columns (ThermoFisher Scientific, MA, USA). After centrifugation with 1200 × *g* for 1 min, the sepharose was reconstituted in 140 µl of coupling buffer and 60 µl crosslinked BSA-MBS-Peptide was applied and incubated overnight at 4°C in an end-over-end shaker. On the next day, the matrix was washed five times with 400 µl coupling buffer and blocked with 400 µl of blocking solution (0.1 M Tris-HCl; 0.5 M NaCl; pH 8.0) for 2 h at room temperature in an end-over-end shaker. Following the blocking of unspecific binding, the matrix was washed with washing solution (27 mM KCl; 43 mM Na_2_HPO_4_; 14 mM KH_2_PO_4_; pH 7.2) until protein detection at 280 nm showed a stable baseline (Äkta Avant25, Cytiva, WA, USA). The matrices were then stored in PBS + 0.02% NaN_3_ at 4°C until usage.

Depletion of samples was carried out using 50 µl of respective matrix slurry in 75 µl micro spin columns (ThermoFisher Scientific, MA, USA). The matrix was washed two times with PBS prior to usage, and 30 µl of serum samples were loaded and incubated for 1 h at room temperature (M1 peptide) or blocked again with peptide microarray blocking buffer overnight before the addition of samples overnight at 4°C (S288 peptide). The depleted samples were obtained as flow through via centrifugation at 1200 × *g*. The matrix with peptide-bound antibodies was washed three times with PBS prior to elution. Elution was done with 30 µl of low-pH IgG-elution-buffer (ThermoFisher Scientific, MA, USA) via spinning at 2500 × *g*. Eluted Antibodies were reconstituted in 3 µl of high salt containing neutralization buffer (ThermoFisher Scientific, MA, USA). The success of the depletion step was controlled by analyzing the flow through with the peptide microarrays. Sera with depleted signals due to M1 or S288 peptide binding, but comparable overall signal patterns were then subjected to the neutralization assay. The relative protection in percent was calculated by setting the average signal of unprotected infected cells to 0% protection, and the average signal of uninfected cells to 100% protection.

### SARS-CoV-2 serology

The humoral immune response was characterized by three different commercial assays. For a highly sensitive detection of past infections, the qualitative Elecsys anti-SARS-CoV-2 Ig assay (Roche; Mannheim, Germany) targeting the viral nucleocapsid protein (NC) was run on the Cobas e411 system (Roche) according to manufacturer's recommendation (cut off ≥1 COI/ml). Analysis of the response against the viral spike protein after infection or vaccination was evaluated by two different quantitative assays the Elecsys Anti-SARS-CoV-2 Spike Ig, (Roche, cut off 0.8 U/ml; Cobas e411 system) and the Liasion TrimericS IgG assay (DiaSorin, cut off ≥33.8 BAU/ml, Liasion XL system) according to manufacturer's recommendation. Samples with titres higher than 250 U/ml (Elecsys Anti-SARS-CoV-2 Spike) or 380 BAU/ml (Liasion TrimericS IgG assay), respectively, were automatically diluted 1:100 or 1:10 in dilution buffer to increase the linear range to 25,000 U/ml or 20,800 BAU/ml respectively.

### Cell culture and viruses

Vero E6 (ATCC CRL-1586) cells were maintained in DMEM medium supplemented with 10% fetal calf serum (FCS) and 2 mM L-glutamine. All incubation of cells and viruses were done at 37°C in a 5% CO_2_ atmosphere. The SARS-CoV-2 strain used in this study is a Zagreb isolate (hCoV-19/Croatia/ZG-297-20/2020, GISAID database ID: EPI_ISL_451934). All work with infectious viruses was performed in a biosafety level 3 facility.

### SARS-CoV-2 infection and neutralization assay

The activity of serum samples on SARS-CoV-2 infection was tested by seeding Vero E6 cells one day before infection at a density of 7×10^3^ cells per well in a 384-well cell culture plate (ThermoFisher Scientific, MA, USA) in 40 µl medium. On the day of infection, 5 µl of serum samples were added to cells and incubated for 1 h. Thereafter, cells were infected with 5 µl SARS-CoV-2 at a multiplicity of infection (MOI) of 0.01 and incubated at 37°C in an IncuCyte S3 live-cell analysis system (Sartorius) for 72 h. The viability of cells was determined 72 h post-infection with the CellTiter-Glo Luminescent cell viability assay (Promega). Cell-Titer-Glo reagents were prepared as per manufacturer's instructions and the reaction was initiated by the addition of 50 µl reagent per well to cells. Plates were incubated in the dark for 10 min at room temperature prior to luminescence measurement. Luminescence was detected using a Synergy HTX Multi-Mode plate reader (Biotek).

### Bioinformatic analysis

Four different classification-based approaches (support vector machines, logistic regression, random forests and lasso feature selection) along with a two-sided Chi-square test with a false discovery rate (FDR) correction were used to identify peptides and the corresponding antibody types that correlate with positive samples. The peptides were then ranked by the number of methods that identified them (Table S1). The number of confirmed methods and available features are indicated in the first two columns: ten peptides were identified by all five methods, eight by four methods, and 15 by three. Using the identified peptides, the predictive power of different classifiers using 10-fold cross-validation as well as the performance evaluation on an isolated test set are provided using F1-score as a performance measure.

## Results

### Peptide microarray-based detection of epitope resolved B-cell responses

Peptides that covered the complete sequences of spike (S), nucleocapsid (N), envelope (E), and membrane (M) proteins of SARS-CoV-2 were SPOT-synthesized as cellulose conjugates [17] and subsequently printed on glass slides as an array using the SC^2^ method [18]. With a length of 15 amino acids per peptide and an offset of three amino acids, S, N, M, and E proteins were represented by 421, 136, 70, and 21 peptides, respectively (Supplemental Data File 1, “Peptides”). The 648 peptides were printed four times per slide with a layout depicted in Figure 1(A). The array was validated with the monoclonal antibody SH2029-G6, that is known to recognize the overall peptide DPSKPSKRSFIEDLLFNKVTLADA. The antibody bound the peptides S270–273 on the peptide microarray, corresponding to the minimal epitope FIEDLLFNK (Supplementary Figure S1); thus, the array mapped the epitope addressed by the monoclonal antibody correctly and precisely. The slides were incubated with 67 heat-inactivated positive and 22 negative serum samples (Table 1). Bound serum antibodies were detected by anti-human IgA, IgG, and IgM antibodies that were coupled to fluorescent dyes. Individual array spots were classified as binding-positive or -negative following a visual inspection. The protocol led to clear responses that reflect specific binding of IgG, IgA, and IgM antibodies to peptides, as exemplified for sample f (6 weeks post-infection) in Figure 1(B).

**Table 1.** Patient and sample information.

Classification	SARS-CoV-2 negative			SARS-CoV-2 positive		
No of individuals	22			36		
No of samples	22			67		
Sex	M	F	n/a	M	F	n/a
	4	5	13	15	17	4
Age	31–39	33–45	–	27–85	16–62	26–30*
Days post-infection	–			4–124	10–117	32–107*
Disease severity						
Asymptomatic				–	1	–
Mild				7	15	8
Moderate				3	–	–
Severe				1	–	–
Critical				1	–	–

Notes: n/a: information not provided; * age and dpi (days post-infection/onset of symptoms) not provided for four individuals.

**Figure 2.**
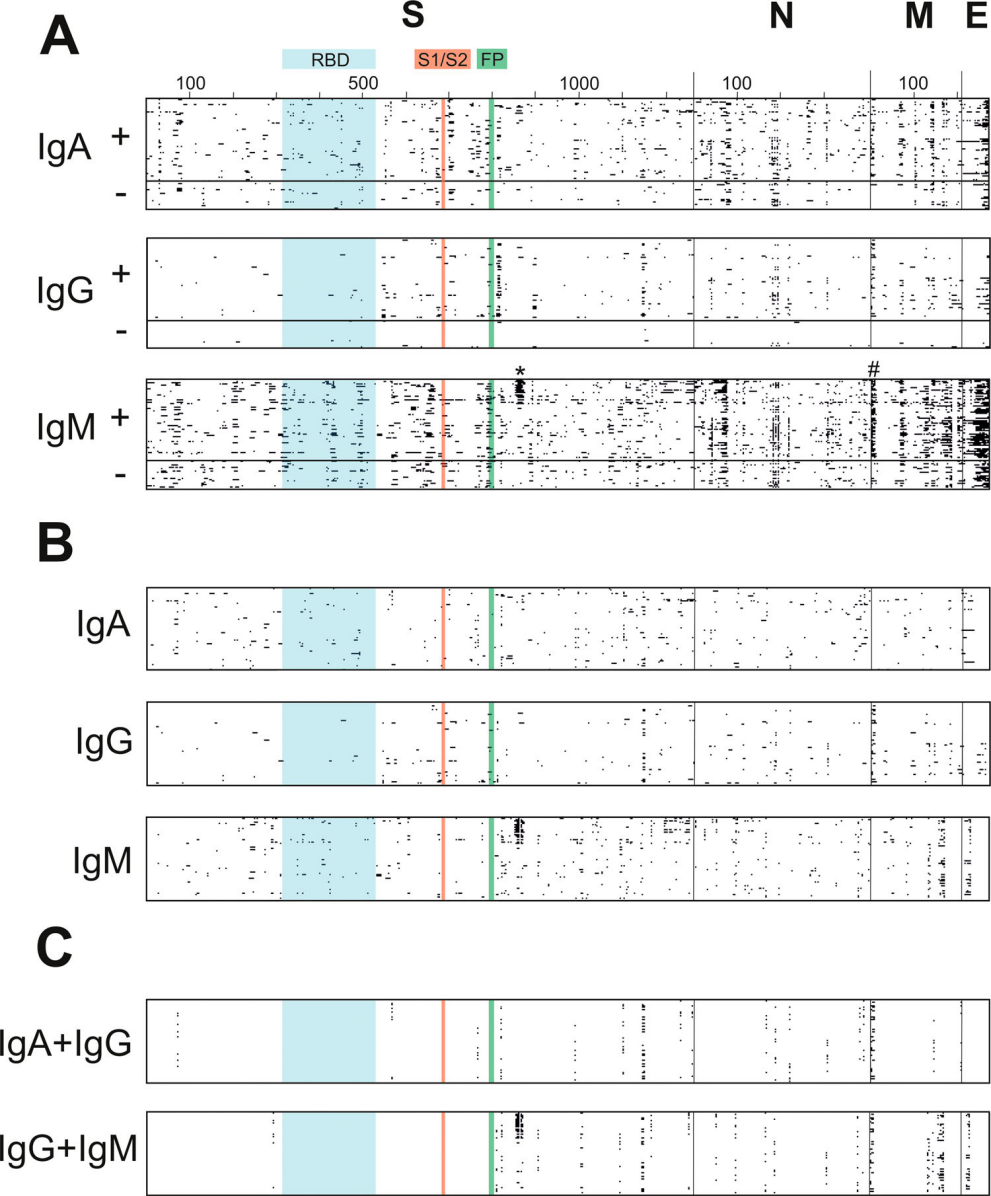
Distribution of positively reacting peptides across SARS-CoV-2 proteins. (A) Each antibody-bound (positive) peptide spot is given as a black square for each isotype (IgA, IgG, and IgM), with 89 rows for 67 SARS-CoV-2 positive (+; upper rows) and 22 SARS-CoV-2 negative samples (−; lower rows) per isotype, and as many columns, as there are peptides on the chip covering the respective protein. Positive and negative samples are separated by a thick horizontal line. Amino acid numbers are indicated for all four proteins investigated; the receptor-binding domain (RBD; aa 319–514) is given in blue, S1/S2 cleavage site (aa 680–685) in red and fusion peptide (FP; aa 788–806) is given in green; borders between the four proteins are indicated by thin vertical lines. Peptides bound to potentially neutralizing antibodies are marked with * and #. (B) SARS-CoV-2 specific positively reacting peptides were observed in data set. The isotype data sets of all 67 positive samples were depleted from spots that were observed in any negative sample, resulting in SARS-CoV-2 humoral immune answers that were specified in this study. (C) Determination of immunodominant epitopes within the specific data set as depicted in Figure 2(B). Specific peptides are marked if they were detected in at least 10% of the samples in individual sets (IgA, IgG, and IgM).

Across all samples, a total of 7852 positively responding peptide spots were observed (Figure 2(A) and Table 2). Among those, IgM antibody binding was more frequent (4992 responses) than the binding of IgA (2116 responses) and IgG isotypes (744 responses). An important feature of the global epitope binding map is that antibodies were detected in both positive and negative sera, but responses in positive samples (6501, mean: 97.0 responses/sample) were more frequent than in negative samples (1351, mean: 61.4 responses/sample) (Table 2 and Supplemental Data File 1). The specificity of IgG responses – mean: 10.3 responses/sample in positive samples vs. mean: 2.5 responses/sample in negative samples – was higher than that of IgA (mean: 26.3 vs. 16.2) and IgM (mean: 60.5 vs. 42.7). These ratios were similar for all four proteins. Most responses were directed against the spike protein, and the overall number of responses decreased in the order S > N > M > E. However, when adjusting for the number of peptides per protein (reflecting protein size), the number of responses per proteins decreased in a reverse order, i.e. E > M > N > S. We attribute the presence of cross-reacting antibodies in SARS-CoV-2 negative patients to a similarity of the relatively short, linear peptides with antigens detected and memorized from former infections, e.g. with other, non-SARS-CoV-2 coronaviruses. In fact, it was recently demonstrated that a large fraction of non-exposed individuals have T-cell reactivity to SARS-CoV-2 peptides, indicating cross-reactivity with existing responses against homologous peptides [21]. On the other hand, the experiment also yielded a series of peptides that were specifically detected in positive and not in negative sera (Figure 2(B) and Supplemental Data File 1).

**Table 2.** Summary data on antibody responses.

Positively responding peptide spots							
Sum	Total	S	N	M	E	Isotype	
7852	2116	974	557	347	238	IgA	All Samples
	744	383	148	155	58	IgG	
	4992	2313	972	952	755	IgM	
6501	1760	822	445	294	199	IgA	SARS-CoV-2 positives
	689	354	135	146	54	IgG	
	4052	1835	787	815	615	IgM	
1351	356	152	112	53	39	IgA	SARS-CoV-2 negatives
	55	29	13	9	4	IgG	
	940	478	185	137	140	IgM	
Signal distribution over proteins (%)							
	Sum	S	N	M	E		
	100	46.0	26.3	16.4	11.3	IgA	All samples
	100	51.5	19.9	20.8	7.8	IgG	
	100	46.3	19.5	19.1	15.1	IgM	
	100	46.7	25.3	16.7	11.3	IgA	SARS-CoV-2 positives
	100	51.4	19.6	21.2	7.8	IgG	
	100	45.3	19.4	20.1	15.2	IgM	
	100	42.7	31.5	14.9	11.0	IgA	SARS-CoV-2 negatives
	100	52.7	23.6	16.4	7.3	IgG	
	100	50.9	19.7	14.6	14.9	IgM	
Signal distribution between positive and negative responses (%)							
	Sum	S	N	M	E		
** **	82.8	84.4	79.9	84.7	83.6	IgA	SARS-CoV-2 positives
** **		92.4	91.2	94.2	93.1	IgG	
** **		79.3	81.0	85.6	81.5	IgM	
** **	17.2	15.6	20.1	15.3	16.4	IgA	SARS-CoV-2 negatives
** **		7.6	8.8	5.8	6.9	IgG	
** **		20.7	19.0	14.4	18.5	IgM	

The global statistical analysis does not take protein structures into account that may make certain epitopes inaccessible. When mapping the responses across the M protein topology [22], we found that its transmembrane regions are hardly targeted by antibodies, whereas the two extraparticular regions were frequently bound (Figure S2). In fact, the extraparticular N-terminus of M constituted one of the most specific epitopes in the study that was addressed in 88% of positive samples. This finding is in line with data from other recent studies [9]. Also, the intraparticular C-terminus of M was frequently targeted. This underlines the importance of intraparticular epitopes for immunogenicity, which was highlighted in previous studies [3,23].

To determine immunodominant epitopes from the peptide microarray data, we have marked epitopes detected with a frequency >10% in the specific data set (Figure 2(C)). Of the 38 specific epitopes found, 19 were directed against the spike protein, whereas no linear epitope was observed within the RBD. Among immunodominant epitopes that were targeted with a frequency > 50% in all samples, a stretch of adjacent peptides covering amino acids 811–827 of the spike protein, a region that is close to the fusion peptide, was detected in 55% of positive (and 32% of negative) samples. This signal was also reported in a longitudinal study using peptide microarrays [9].

The longitudinal sampling for some patients allowed to closely follow the maturation of the immune response. Patient g developed first IgM and IgA antibodies after two weeks post-infection (wpi), while IgG responses occurred only after four wpi or later (Figure S3). This is in line with a commercial IgG chemiluminescence immunoassay (Liasion XL, Diasorin), which also showed a negative result for the spike protein at two wpi for the respective patient (Supplemental Data File 1). Some antibodies were not retained over time, for example, IgA's addressing peptides S258–S260 at four wpi were not observed at 13 wpi. We note that antibodies addressing peptides S286–S289 appeared first at 13 wpi, an epitope that was particularly important for immunogenicity and neutralization across the whole sample set (see below). This epitope was found in every late positive serum sample (12 wpi or later), and its detection in the IgM subclass suggests that antibodies against it were continuously developed.

We used machine learning approaches to determine a combination of peptides with high predictive value for differentiating positive and negative samples, e.g. to select peptide components for a diagnostic SARS-CoV-2 test. We used four different classification-based feature selection methods (support vector machines, logistic regression, random forests, and lasso feature selection) to identify peptides and the corresponding antibody types that correlate with positive samples and in addition performed a two-sided Chi-square test with a false discovery rate (FDR) correction and alpha = 0.05. The peptides were then ranked by the number of methods that identified them. Ten peptides were identified by all five procedures (Table S1), eight by four procedures, and 15 by three. Using the identified peptides, we next tested the predictive power of different classifiers using 10-fold cross-validation, as well as evaluated performance on another test set using the F1-score as a performance measure. Among these, logistic regression generally performed better than support vector machines or random forests (Table S1). Reducing the number of peptides further improved the nested F1-score, with the highest score of 0.97 ± 0.04 achieved when using the top 33 peptides (i.e. peptides detected by at least three methods), which may be considered as an upper bound for performance on novel samples. This classification showed a false positive rate of 0.02 and a false negative rate of 0.15.

We next investigated the question whether there is a link between immune responses and disease severity. Samples of infected patients were grouped into the two categories “asymptomatic/mild” (*n* = 55) and “moderate/severe/critical” (*n* = 12) disease, and Fisher's exact test with an FDR correction for multiple testing was conducted for each peptide, to check whether binding to it was more likely in any of the two categories. Binding to nine peptides, i.e. S81, S82, S83, S227, S271, S272, S382, S383, N74, was significantly (*p* < 0.05) more frequent in samples from patients with moderate/severe/critical disease (Table 3). In contrast, peptides that were bound more frequently in cases of asymptomatic/mild disease were not detected. For example, IgG antibodies against S382–S383 (aa 1144–1181) were produced in 92% (11/12) of moderate/severe/critical cases, but in solely 7.3% (4/55) of mild/asymptomatic cases. Remarkably, N74 showed 50% (6/12) IgG responses in moderate/severe/critical cases and was completely absent (0/55) in the asymptomatic/mild group. Moreover, IgG antibodies directed against S271–272 (aa 811–827), binding in close proximity to the fusion peptide, which spans amino acids 788–806, were detected in 83.34% (10/12) of moderate/severe/critical and 45.45% (25/55) of asymptomatic/mild cases, respectively. In addition to IgG isotypes, the peptides S81–S83 and S227, enriched in moderate/severe/critical cases [41.67% (5/12) vs. 1.8% (1/55) and 25.0% (3/12) vs. 1.8% (1/55), respectively], belong to the IgA isoform. The finding that most of the significantly binding peptides come from the spike protein is also reflected by the fact that the share of binding events to S was higher in moderate/severe/critical cases compared to asymptomatic/mild cases (Table S3). This was observed in early as well as late phases post-infection. The peptides increasingly bound in more severe cases might reflect an exacerbated immune response that is a hallmark of severe COVID-19. Notably, all nine significantly enriched peptides are predicted as immunogenic by the in silico sequential B-cell epitope predictor BepiPred 2.0 [24]. Although larger sample sets with more detailed patient classifications are required to assess the value of the peptides for prognosing disease severity, the detailed peptide microarray analysis demonstrated its value as a discovery tool for biomarker candidates.

**Table 3.** Peptides bound more likely in patients with moderate/severe/critical disease.

		Asymptomatic/mild		Moderate/severe/critical	
Peptide	*p*-Value	Bound	Unbound	Bound	Unbound
S81	.0133	0	55	5	7
S82	.002	0	55	6	6
S83	.0321	1	54	5	7
S227	.0321	1	54	5	7
S271	.0156	17	38	11	1
S272	.0156	17	38	11	1
S382	.0001	11	44	12	0
S383	.0005	14	41	12	0
N74	.0204	4	51	7	5

### Bioinformatic detection of antibodies contributing to neutralization capabilities of sera

**Figure 3.**
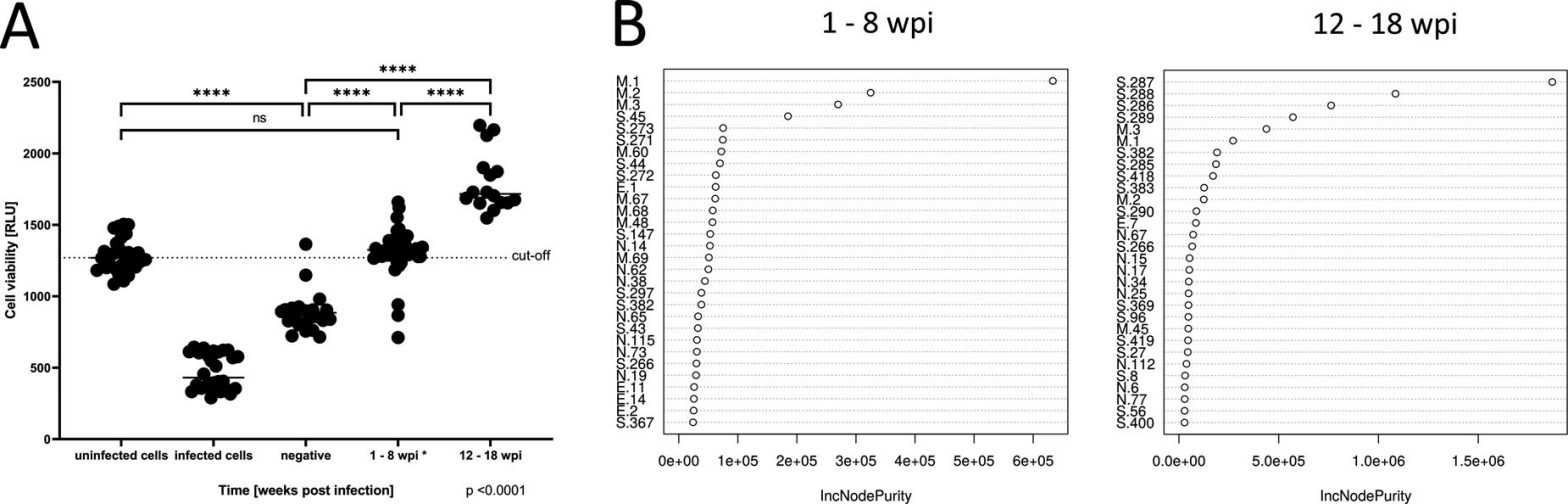
Identification of peptide epitopes contributing to neutralizing capabilities of serum samples. (A) All serum samples (*n* = 89) were tested for their ability to neutralize SARS-CoV-2 infectivity in Vero E6 cells using an ATP-based viability assay. The mean Relative Light Unit (RLU) level of uninfected cells is visualized as a dotted line, serving as a cut-off for complete protection from viral infection. Positive samples were divided into two groups of 1–8 weeks post-infection (wpi) (*n* = 51) and 12–18 wpi (*n* = 16). *No significant differences were observed dividing the group 1–8 wpi into 1–4 wpi and 5–8 wpi (data not shown). ****p < .0001. (B) Detection of peptides contributing to neutralization by a random forest analysis for sera from 1–8 wpi and 12–18 wpi. Peptides are ranked via the mean increase in node purity (IncNodePurity) when splitting on the variable, averaged over all decision trees in the random forest.

Next, we assessed the ability of sera to neutralize SARS-CoV-2 in a cellular assay. For this purpose, the pathogenic effect exerted upon the infection of Vero E6 cells with SARS-CoV-2 (isolate hCoV-19/Croatia/ZG-297-20/2020) in the presence of sera at a 1:10 dilution was monitored by an ATP-based assay (Supplemental Data File 1). We observed that the neutralizing capacities of positive sera sampled 12–18 weeks post-infection (wpi) were significantly higher than those of sera 1–8 wpi (1796 U vs. 1312 U, *p* < .0001), whereas the cell viability upon addition of negative sera was lowest (884 U, *p* < .0001) (Figure 3(A)). These data demonstrate that the positively diagnosed patients in the study indeed elicited a protective immune response.

**Figure 4.**
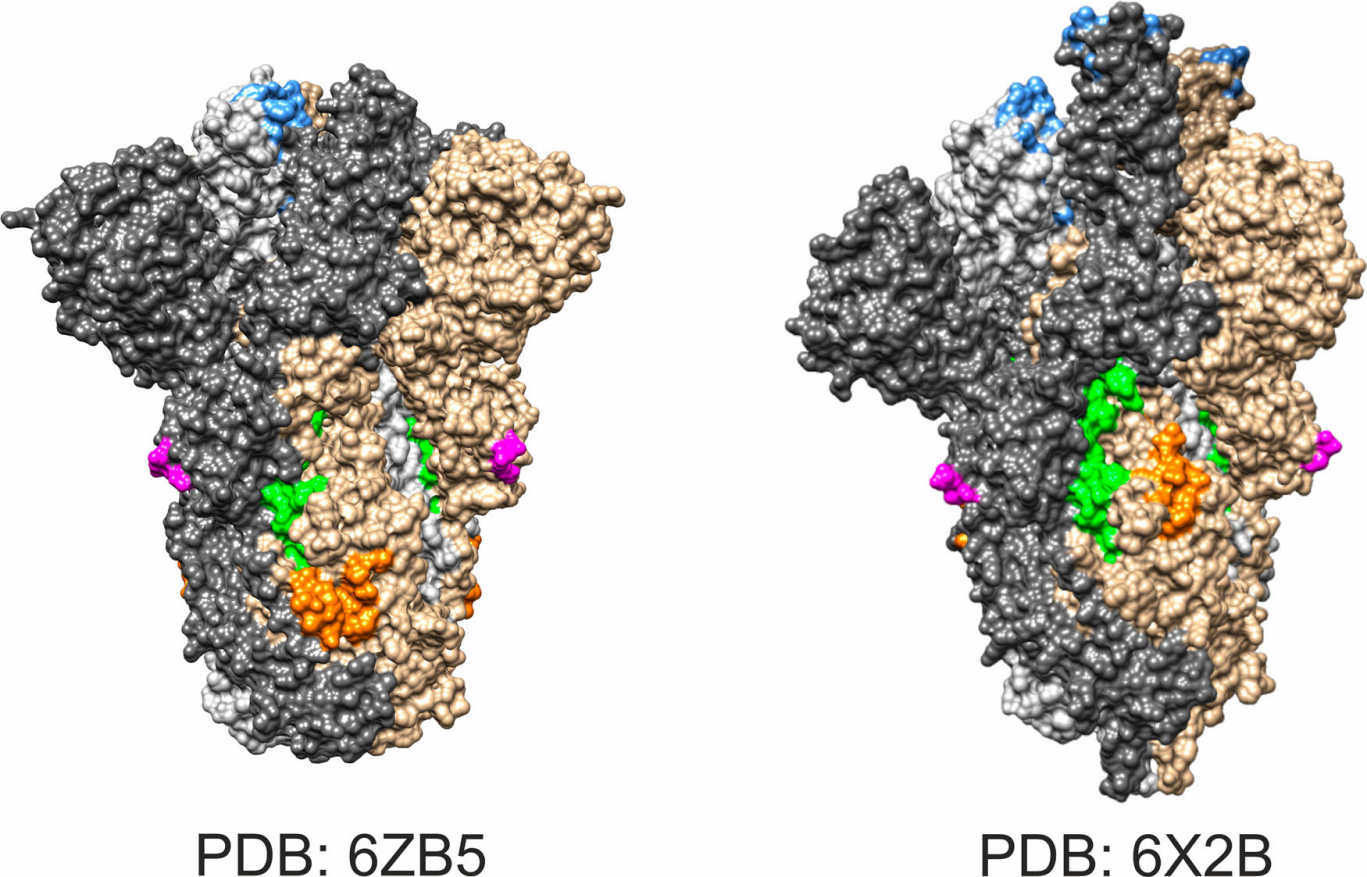
Mapping of neutralizing antibody binding region on the spike protein. Projection of antibody target site (aa 853–879; green) onto spike trimer (Chain A, B, and C in grey, dark grey, and brown) in closed (PDB: 6ZB5) and open state (6X2B). The binding domain is close to the fusion peptide (aa 788–806; orange). The S1–S2 cleavage site is disordered in crystal structures, but adjacent amino acids were marked in magenta. Amino acids relevant for ACE2 binding are given in blue.

Although the overall neutralization results from a polyclonal and individually unique B-cell response signature, we wondered whether there are individual epitopes whose detection correlated with a protective effect. For each peptide in the microarray, we used a one-sided Mann–Whitney U test with an FDR correction for multiple testing to probe if neutralization values in samples where the peptide was bound by any of the three Ig isotypes were higher than in samples with an unbound peptide. This identified 21 significant peptides (*p* < .05, Table S2), mainly located in the S protein (S96, S284, S285, S286, S287, S288 S289, S290, S389, S400, S411, S416, S417, S418, S419), but also in the N protein (N1, N25), M protein (M1, M2, M3) and E protein (E7). To identify the peptides with the strongest predictive value of neutralization capacity when considered in combination with others at different times after infection, we used a random forest analysis for groups of early samples (1–8 wpi) and later samples (12–18 wpi), respectively. The random forest assesses the importance of all peptides for predicting neutralization by calculating the increase in node purity (IncNodePurity) from splitting on the variable, averaged over all decision trees in the random forest. For positive sera 1–8 wpi, the four peptides M1, M2, M3, and S45 were the strongest predictors of neutralization with IncNodePurities of 185 K or higher (Figure 3(B)). Among these, S45 is the only peptide not previously identified by the one-sided Mann–Whitney U test, as its binding seems to be negatively correlated with high neutralization. Interestingly, the list of top-ranked peptides in the random forest analysis changed for a longer sampling timeframe of 12–18 wpi: The top five peptides that stood out from the rest were S287, S288, S286, S289 and M3. With an IncNodePurity of >438 K, they all contributed more strongly to protection compared to the top peptides for 1–8 wpi. The N-terminal peptides of the M protein were detected in both timeframes with high frequencies with all Ig isoforms. In contrast, the S286–S290 region of the spike protein was hardly bound 1–8 wpi, but the immune response against this region evolved at 12–18 wpi, and unexpectedly, it was particularly strong for IgM (Figure S4). A mapping of the S286–S290 region onto the closed (PDB: 6ZB5) and open state (6X2B) 3D structures of the trimeric spike protein visualized that the epitope is located in close proximity of the fusion peptide region (Figure 4), distant from the S1–S2 cleavage site or the ACE2 (RBD). Access to this region starting at Q853 does not appear to be hampered by glycosylation sites, as the closest known N-glycosites were located inside the fusion peptide N801 or between the two heptad repeat N1073 [25].

### Antiviral effects of antibodies binding M1 and S288

**Figure 5.**
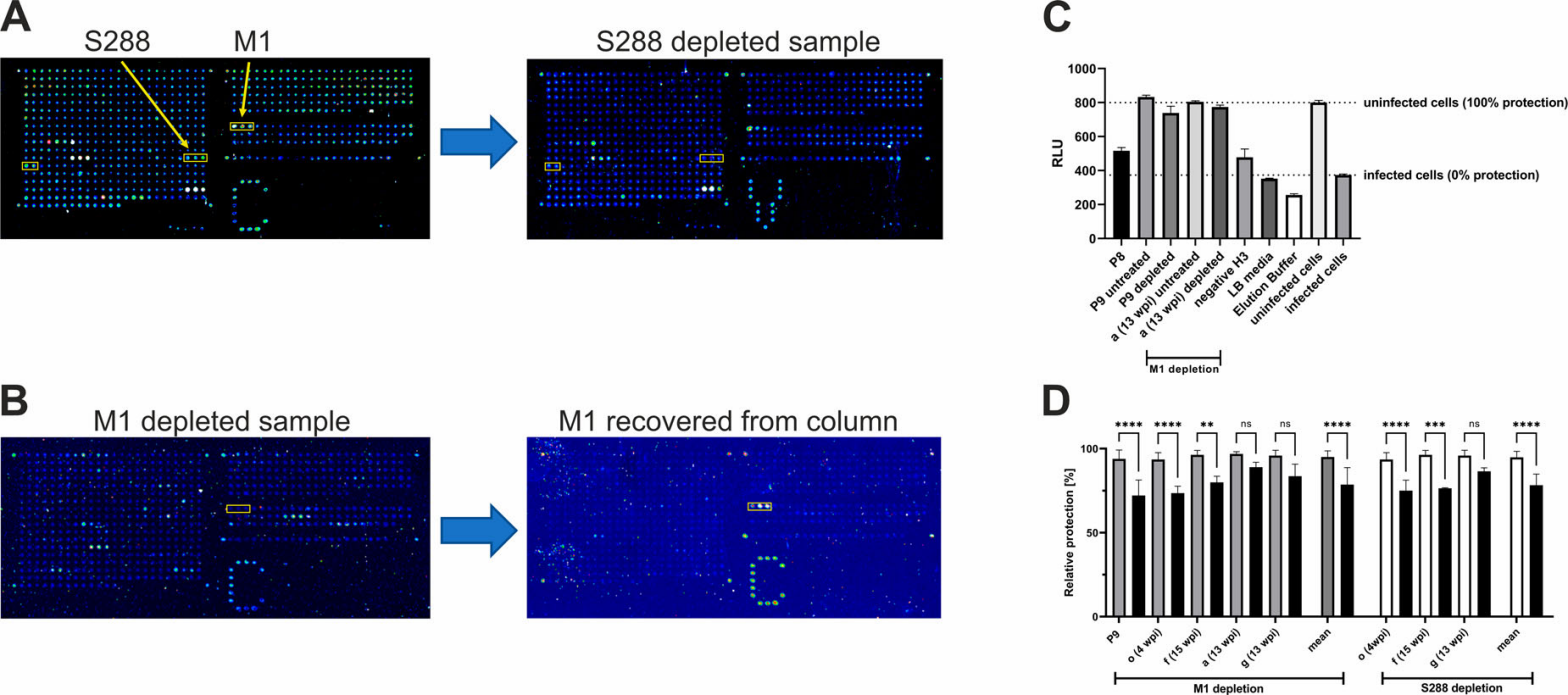
Depletion of antibodies against M1 and S288 impairs viral neutralization capacity. (A and B) Control of depletion efficacy by peptide microarrays. (A) Serum f (15 wpi) was depleted from S288 binding antibodies by an S288-functionalized column. Peptide microarrays before (left) and after (right) the depletion step exhibit lower signals in the S288 region. (B) Peptide microarrays after purification of serum a (6 wpi) by an M1-functionalized column (left) and after re-elution from the column (right). A depletion (left) and recovery (right) of M1-binding antibodies is visible. Positions of peptides M1–M3 and S286–S290 on the array are marked by yellow boxes. (C) Neutralization capacity of sera following M1 depletion. Viability of Vero E6 cells is assessed by their ATP content and detected in relative luminescence units (RLU). Uninfected cells were viable, whereas infected cells showed cytopathic effects, leading to lower RLU reads (two right bars). The samples P9 and a (13 wpi) showed a slight reduction of neutralizing capacity compared to the untreated control. All samples were diluted 1:10 and measured four times per experiment. (D) Relative protection as measured in C. As more samples for M1 depletion were available we measured five samples (untreated controls: grey bars vs. depleted samples: black bars) and three of them with known reactivity against S288 were used for depletion (untreated controls: white bars). The obtained readouts showed comparable results ranging from 7.88% to 21.82% with both significant outcomes for mean values (*p* < .0001).

We aimed to validate experimentally whether the N-terminal epitope of the M protein (M1–M3) and the newly identified region on the S protein (S286–S290) contributed to neutralization. For this purpose, antibodies binding to peptide M1 or to peptide S288 were depleted from the sera in separate experiments by capturing them on sepharose columns that were functionalized with the respective synthetic peptides. The effectiveness of the depletion steps was proven via peptide microarray analysis. Signals due to binding of antibodies against peptides M1 or S288 disappeared, while signals induced by other reactive antibodies prevailed (Figure 5(A,B)). In turn, purified antibodies binding specifically to M1 could be recovered following elution from the column (Figure 5(B)). The neutralization capacity of the depleted sera on the infection of Vero E6 cells with SARS-CoV-2 was compared to that of the native, untreated sera. Due to the very limited amount of samples, only a single dilution ratio of 1:10 was investigated for a selection of sera. For example, serum P8 and the negative serum H3 showed only moderate virus inhibition, as expected from previous neutralization experiments, whereas sera P9 and a (13 wpi) completely neutralized the virus (Figure 5(C)). For five selected sera with high neutralization capacities, named P9, o, f, a and g, the depletion of antibodies binding to M1 led to the loss of neutralizing activity against SARS-CoV-2, ranging from 7.88% to 21.82% with a mean of 16.42% (Figure 5(D)). The effects were non-significant for a and g, but significant for P9, o and f, and for the mean of all samples (*p* < .0001). Three sera depleted from S288-binding antibodies exhibited neutralization capacities that were decreased by 9.37%–19.93% with a mean of 16.60% (*p* < .0001). In contrast, there was no decrease in neutralization when the samples were bound to and eluted from a column that was functionalized with a randomly selected peptide. This finding shows that the decrease in neutralization was not due to an unspecific binding to the column. The neutralization capacity of the eluate from S288 or M1 bound sera could not be determined, unfortunately, because the elution buffer itself was toxic to cells.

The experiments demonstrate that peptides S288 and M1, predicted as relevant for neutralization by the random forest model, indeed possess a neutralizing activity against SARS-CoV-2 infection.

## Discussion

This study reports an epitope-resolved analysis of the B-cell response of patients infected with SARS-CoV-2 by a combination of peptide microarrays and machine learning methods. The peptides represent the target regions (epitopes), i.e. those parts of the whole antigenic protein, which are detected by antibodies with complementary binding regions. The binding patterns of antibodies from sera of 67 positive and 22 control patients were complex and showed responses across all Ig isotypes in both groups. The presence of cross-reacting antibodies in SARS-CoV-2 negative patients is attributed to a similarity of the relatively short, linear peptides with antigens detected and memorized from former infections, e.g. with other, non-SARS coronaviruses. This observation is substantiated by previous studies [26], e.g. those of Mishra et al. [11], Ladner et al. [10]. and Heffron et al. [15], who reported maps of cross-reactivity with endemic human coronaviruses throughout the SARS-CoV-2 proteome, mostly looking at IgG. It was also demonstrated that a large fraction of non-exposed individuals have T-cell reactivity to SARS-CoV-2 peptides, indicating cross-reactivity with existing responses against homologous peptides [21]. Given the timeframe of sampling, all infections were probably caused by the initial SARS-CoV-2 variant reaching Europe. Thus, the responses stem from a relatively homogeneous cohort with respect to the pathogen; on the other hand, a limitation of the study is that it did not account for virus evolution by capturing variant-specific responses.

We found more binding events for IgM than for IgA or IgG, which was also true when epitopes addressed in negative control sera were removed from the data set to obtain SARS-CoV-2 specific reads (compare Figure 2(A,B)). In contrast to other studies reporting a decline in neutralizing ability over time [27], we observed increasing levels of neutralization until week 18 post-infection. This may reflect that antibodies undergo a constant refinement of their targeted epitopes as B-cells mature over time. Interestingly, newly emerging SARS-CoV-2 specific IgM responses were also found at late sampling timepoints (12–18 wpi). It is noteworthy that most samples (78.6%, 11/14) in this set were derived from mild infections and had the highest neutralization values. It might be possible that SARS-CoV-2 is persistent in low copy numbers [28,29], thereby leading to constant B-cell answers; however, a larger number of samples needs to be investigated to draw firm conclusions on the IgM response dynamics.

It is possible that the peptide microarray missed conformational epitopes, as it comprised only short, linear peptides. For comparison, standard immunoassays detecting IgGs against whole proteins were performed, which also captured conformational epitopes. These assays showed false-negative rates of 21.7%, 9.5% and 15.4% for the spike, the RBD and nucleocapsid in the longitudinal sample set, respectively (Supplemental Data File 1). Some of the false-negative detections are probably due to the early timepoint of sampling, where the sensitivity of the immunoassay is not optimal yet. In the context of this study, we conclude that the false negative rates of the peptide array coupled to machine learning procedure based only on linear epitope information were comparable to those of a standard immunoassay. From the set of peptides that were specifically bound by SARS-CoV-2 positive samples, a diagnostic multiplex peptide-based antibody test would need at least 12 different peptides (Supplemental Data File 1). To achieve 100% specificity and sensitivity in such test, all Ig isoforms need to be tested.

The high specificity of the spike protein at amino acids (aa) 570–590 found in the IgG set by Heffron et al. could not be confirmed in our study, as the corresponding signal was only observed in 6% (4/67) of the positive samples. Moreover, peptides starting at aa 570 showed antibody binding by the IgA class in our negative sample set. On the other hand, strong responses to the N-terminal region of the M protein were found in both studies. Overall the high complexity of peptide array manufacturing and testing does not render this approach competitive for a simple, binary diagnostic application, in particular when compared to optimized multiplexed immunoassays [30,31], however, we note that a transfer of findings from discovery microarrays to more common platforms has been shown to be feasible by us [3]. Instead, the ability to reveal functionally relevant epitopes is a strength of the microarray technology. This is demonstrated by pinpointing peptides associated with disease severity. Peptides S271–S272 were reported as immunogenic and also neutralizing in previous studies [10,11,13], interestingly, they were also more frequently detected in severely ill, late patients than in patients with mild disease in the study of Mishra et al., albeit a statistical significance was not given. Because the number of severe cases was small in this study, peptide candidates need to be confirmed in a larger, independent sample set, that includes longitudinal time points to evaluate development, before being functionally linked, e.g. to autoimmune reactions.

We also explored the presence of epitope signatures by linking them to neutralizing effects of sera. From the predicted list of contributors, we have validated two candidate peptides experimentally by depleting the antibodies that bind to them from sera. Remarkably, the depletion of binders to a single peptide was sufficient to lower the neutralizing capability of the serum by ca. 16% on average.

The N-terminus of the SARS-CoV-2 M protein has been observed to exert a strong B-cell response by Heffron et al. [15], and our data suggest that this region of M might be a promising target for vaccine development. The same is true for the region covered by peptides S286–S290 of the S protein located close to the fusion peptide. Due to the functional importance of the fusion region for cellular infection, a neutralization activity of antibodies binding nearby appears plausible. In fact, it has been recently noticed that the open conformation of the S protein leads to increased accessibility and high calculated epitope scores for aa residues 850–854, that are located adjacent to the S286–S290 region (aa 853–879) [32]. The experimental evidence provided in this study suggests that this linear epitope is an alternative to the (RBD) domain that currently receives the most attention in vaccine and therapeutic antibody design.

Finally, peptide microarrays were shown to be a powerful tool that can be used to study and compare B-cell responses to different variants of concern of a given pathogen, thereby offering insights into differences and overlaps in response profiles. In particular, information on cross-neutralization may inform the selection of new vaccine target sites.

In summary, we demonstrate how a combination of high-resolution peptide microarrays, machine learning methods and infection assays provides detailed insights into the epitope-resolved immune response against SARS-CoV-2. In spite of the complex nature of these responses, correlating single components with functional parameters like disease severity and neutralization capabilities was possible, and enabled the suggestion of little explored antigenic targets that need to be further validated in subsequent studies.

## Supplementary Material

Supplemental Material

Supplemental Material
