# Supplementary material for: Peptide microarrays coupled to machine learning reveal individual epitopes from human antibody responses with neutralizing capabilities against SARS-CoV-2
Source: Emerg Microbes Infect. 2022 Apr 11;11(1):1037–48. doi: 10.1080/22221751.2022.2057874 (PMC9009950; doi:10.1080/22221751.2022.2057874)
Supplement: Supplemental Material [file TEMI_A_2057874_SM6108.docx]

**Supporting Information**

**Peptide microarrays coupled to machine learning reveal individual epitopes from human antibody responses with neutralizing capabilities against SARS-CoV-2**

Table S1**.** Performance of single peptides in the prediction of positive samples across five methods.

| **number of features** | | **classifier** | **nested f1_pos** | **test f1_pos** | **nested f1_macro** | **test f1_macro** | Nested CV-FPR | Nested CV-FNR |
| --- | --- | --- | --- | --- | --- | --- | --- | --- |
| 5 methods | 10 | lsvm | 0.87 ± 0.12 | 0.77 | 0.8 ± 0.16 | 0.58 | 0.12 | 0.30 |
|  | 10 | lr | 0.9 ± 0.13 | 0.93 | 0.84 ± 0.18 | 0.8 | 0.07 | 0.00 |
|  | 10 | rf | 0.91 ± 0.09 | 0.93 | 0.85 ± 0.15 | 0.8 | 0.08 | 0.25 |
| >=4 methods | 18 | lsvm | 0.89 ± 0.1 | 0.86 | 0.85 ± 0.15 | 0.68 | 0.08 | 0.10 |
|  | 18 | lr | 0.9 ± 0.11 | 0.86 | 0.83 ± 0.16 | 0.68 | 0.08 | 0.00 |
|  | 18 | rf | 0.95 ± 0.06 | 0.93 | 0.9 ± 0.11 | 0.8 | 0.08 | 0.20 |
| >=3  methods | 33 | lsvm | 0.91 ± 0.08 | 0.86 | 0.84 ± 0.14 | 0.68 | 0.12 | 0.15 |
|  | **33** | **lr** | **0.97 ± 0.04** | **0.86** | **0.92 ± 0.09** | **0.68** | **0.02** | **0.15** |
|  | 33 | rf | 0.93 ± 0.08 | 0.93 | 0.87 ± 0.14 | 0.8 | 0.08 | 0.15 |
| >=2  methods | 54 | lsvm | 0.94 ± 0.04 | 0.93 | 0.89 ± 0.08 | 0.8 | 0.17 | 0.05 |
|  | **54** | **lr** | **0.95 ± 0.07** | **1.0** | **0.93 ± 0.09** | **1.0** | **0.12** | **0.20** |
|  | 54 | rf | 0.92 ± 0.09 | 0.93 | 0.85 ± 0.16 | 0.8 | 0.05 | 0.15 |
| >=1 methods | 88 | lsvm | 0.89 ± 0.1 | 0.93 | 0.78 ± 0.19 | 0.8 | 0.18 | 0.15 |
|  | **88** | **lr** | **0.96 ± 0.06** | **1.0** | **0.94 ± 0.09** | **1.0** | **0.13** | **0.15** |
|  | 88 | rf | 0.92 ± 0.1 | 0.93 | 0.84 ± 0.17 | 0.8 | 0.12 | 0.15 |
| All features | 1944 | lsvm | 0.86 ± 0.14 | 0.93 | 0.76 ± 0.2 | 0.8 | 0.17 | 0.25 |
|  | 1944 | lr | 0.88 ± 0.07 | 0.93 | 0.77 ± 0.12 | 0.8 | 0.13 | 0.30 |
|  | 1944 | rf | 0.88 ± 0.09 | 0.93 | 0.71 ± 0.2 | 0.8 | 0.08 | 0.50 |

lsvm = linear support vector machine; lr = logistic regression; rf = random forest; nested CV-FPR. The top-three settings are highlighted in bold, and the best performing setting is marked in red.

Table S2**.** Antibody-bound peptides associated with significantly higher neutralization values.

| **peptide** | **p-value^a)^** | **importance^b)^ 1-8 wpi** | **importance 12-18 wpi** |
| --- | --- | --- | --- |
| S96 | 0.04640 | 14711.6 | 47269.7 |
| S284 | 0.01244 | 0.0 | 17436.5 |
| S285 | 0.00003 | 15555.5 | 185843.7 |
| S286 | 0.00002 | 4101.5 | 762775.8 |
| S287 | 0.00001 | 2530.3 | 1871241.8 |
| S288 | 0.00005 | 2863.6 | 1085452.9 |
| S289 | 0.00003 | 0.0 | 571456.5 |
| S290 | 0.00141 | 2.4 | 87445.8 |
| S389 | 0.04172 | 869.7 | 9349.9 |
| S400 | 0.03268 | 32.6 | 28030.6 |
| S411 | 0.04049 | 13704.1 | 1633.7 |
| S416 | 0.01708 | 0.0 | 5403.3 |
| S417 | 0.04172 | 28.7 | 8542.8 |
| S418 | 0.00869 | 34.7 | 170970.0 |
| S419 | 0.03268 | 1275.2 | 45671.1 |
| N1 | 0.02399 | 5.6 | 455.6 |
| N25 | 0.01707 | 4933.8 | 48246.7 |
| M1 | 0.00869 | 633369.5 | 271141.2 |
| M2 | 0.01413 | 324778.2 | 124343.8 |
| M3 | 0.00090 | 269674.6 | 438437.1 |
| E7 | 0.03328 | 18739.7 | 84961.1 |

a) all peptides with a p-value < 0.05 are listed. b) Variable importance as measured by the increase in node purity (IncNodePurity) for predicting neutralization calculated via random forest models.

Table S3. Distribution of antibody detection across the four structural proteins during asymptomatic/mild (am) and moderate/severe/critical (msc) disease progression.

| **Distribution across structural proteins^1^** | | | |
| --- | --- | --- | --- |
| **protein** | **IgA (am / msc)** | **IgG (am / msc)** | **IgM (am / msc)** |
| S | 44.68% / **54.86%** | 45.45% / **62.61%** | 44.92% / **47.57%** |
| N | 25.67% / 23.71% | 22.39% / 14.29% | 19.47% / 19.10% |
| M | 16.88% / 16.00% | 22.84% / 18.07% | 20.73% / 16.22% |
| E | 12.77% / 5.43% | 9.31% / 5.04% | 14.87% / **17.12%** |

1. Relative distribution, sums up to 100% for am and msc, respectively. Higher occurrence of antibody detection in the msc group is indicated in bold.


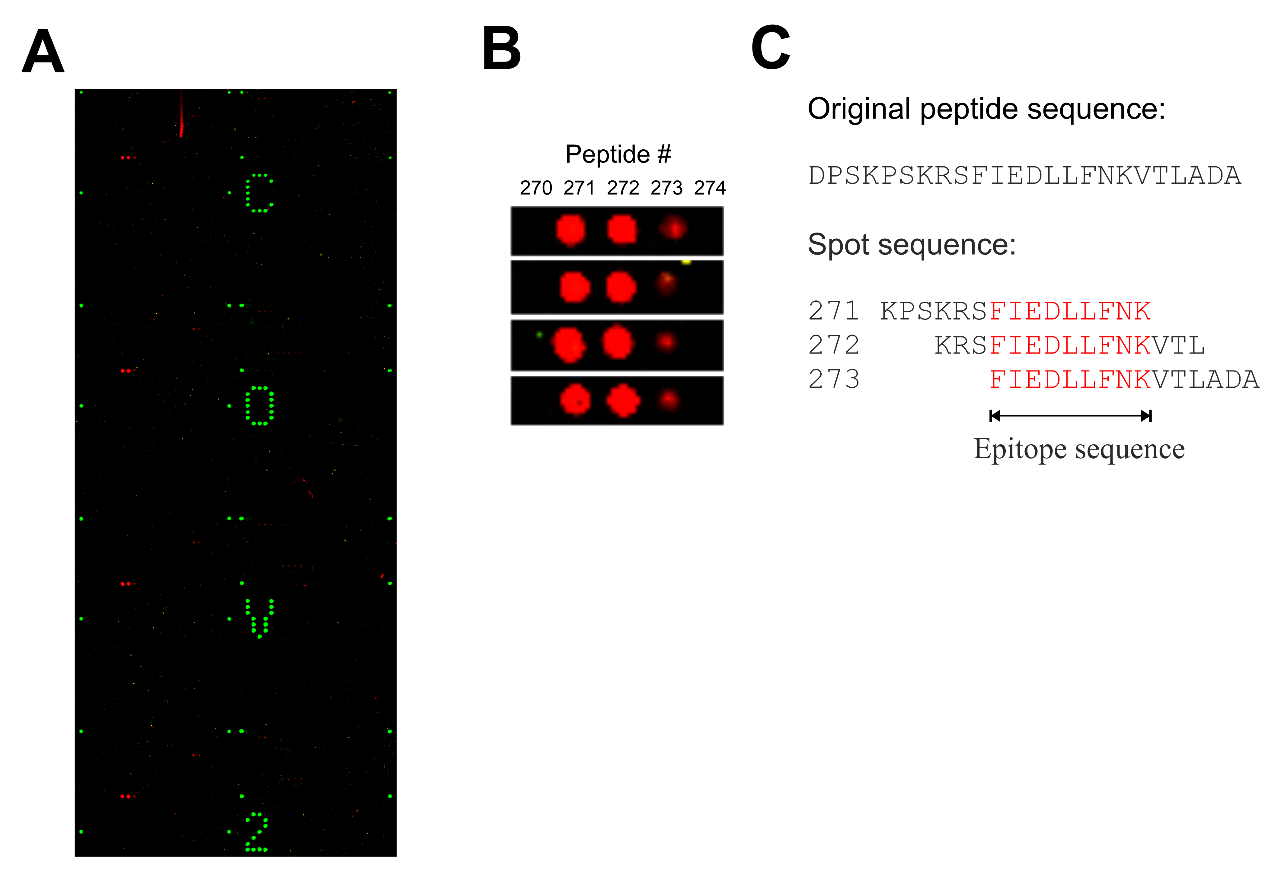


Figure S1. Validation of peptide microarrays. The microarray was incubated with the monoclonal IgG antibody SH2029-G6 known to bind the peptide sequence DPSKPSKRSFIEDLLFNKVTLADA. (A) The bound monoclonal antibody was detected as red spots in equal amounts on each of the four subgrids (C, O, V and 2, surrounded by stained biotin in green) onto the same slide. (B) Magnified reaction pattern of bound monoclonal antibodies on peptide spots S271-S273. (C) The minimal epitope sequence derived from peptides 271-273 is marked in red.


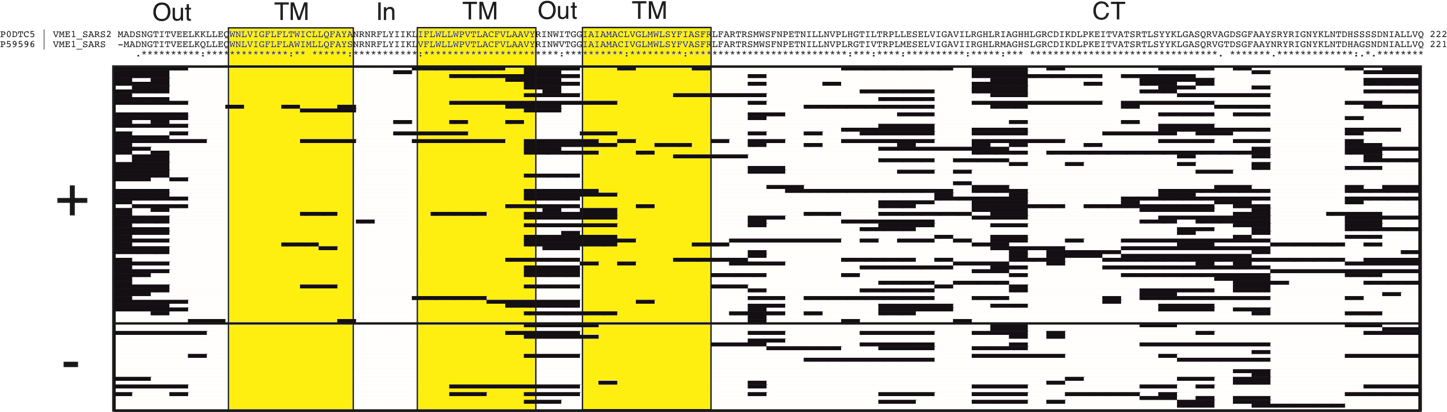
Figure S2. Overlay of M protein topology and peptide microarray binding sites**.** M protein amino acid sequences from SARS-CoV and SARS-CoV-2 virus (Uni-ProtKB: P59596 and P0DTC5. respectively) were aligned and showed 89.2% identity (top). Transmembrane (TM) regions are marked in yellow. Observed bound primary antibodies combined for all isotypes (IgA/IgG and IgM) are represented as black boxes. SARS-CoV-2 positive (+) and negative (-) samples are indicated on the left. TM regions and the short intra virion (In) region between TM 1 and 2 exhibited less antibody binding. Outer virion (Out) parts of the M protein as well as the internal cytoplasmic tail (CT) showed high immunogenicity.


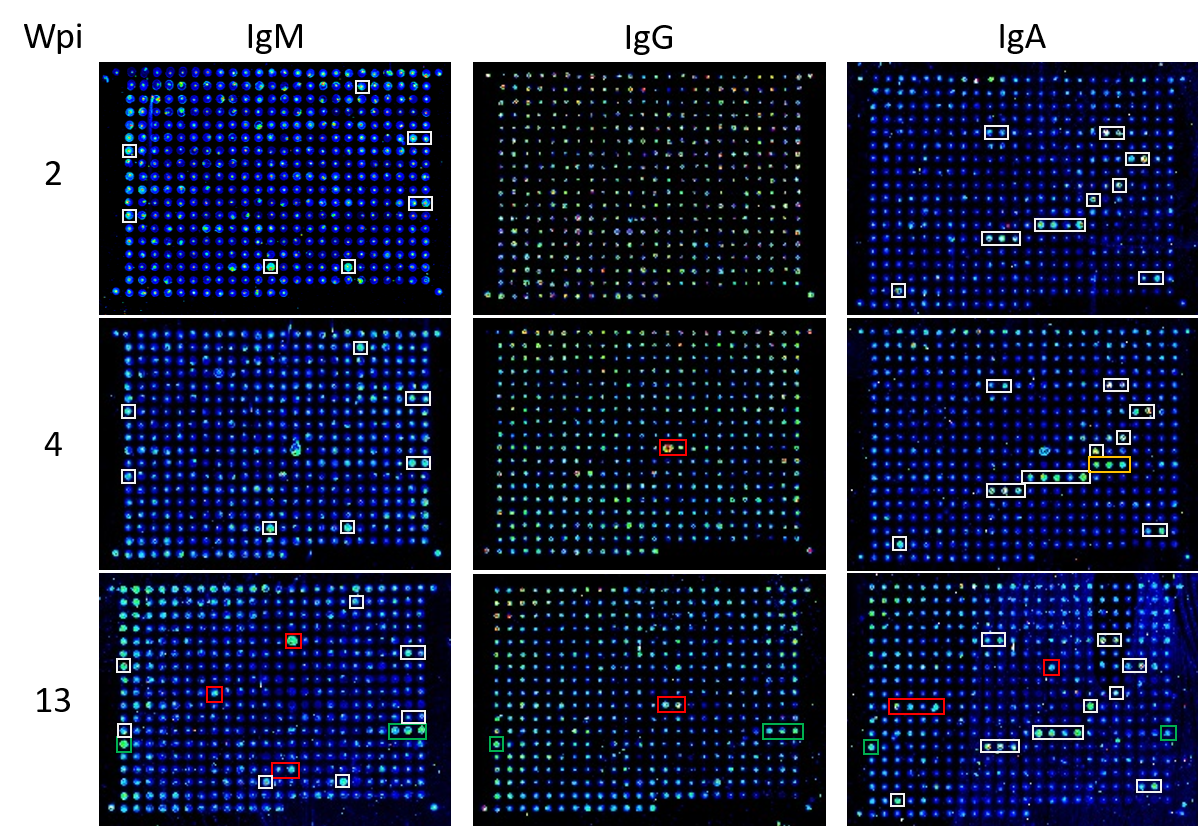


Figure S3. Antibody development of patient g against the spike protein. The development of antibody responses against the spike protein found in samples of patient g at time points 2. 4 and 13 wpi is shown as original peptide microarray data displayed in 16-bit pseudocolor. Bound antibodies of each isotype (IgM. IgG and IgA) are represented by lighter colored (green to white) and thicker dots over background (smaller dots; blue to black range). Antibodies found at 2 wpi and observed over all time points are surrounded by white boxes. New appearing antibodies are surrounded by red boxes. IgA antibodies targeting peptides S258-260 were found only in sample 4 wpi (yellow box). Antibodies targeting the epitope S286-289 appeared at 13 wpi in all isotypes (green box).


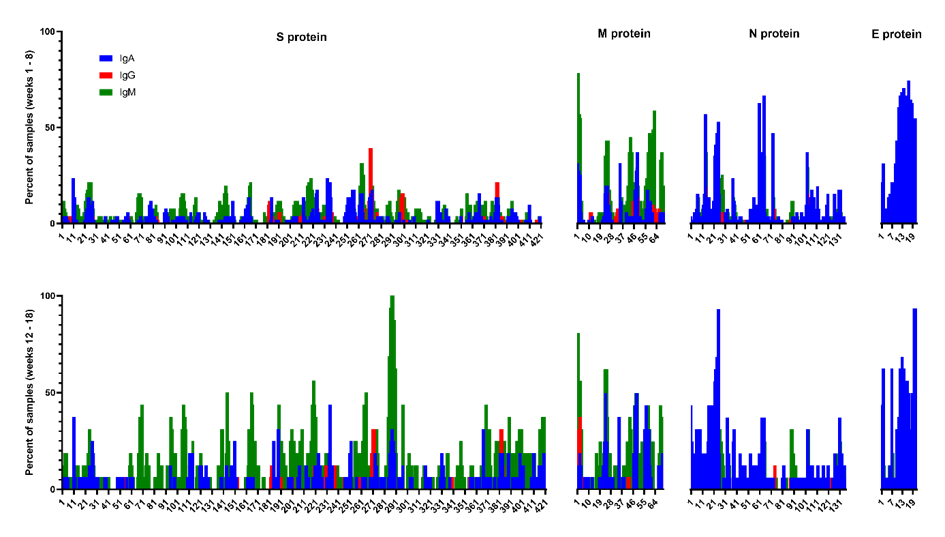


Figure S4. Frequency of occurrence of positively reacting peptides in S.M. N and E proteins. The frequency of occurrence of positively reacting peptides is plotted vs. the peptide number across spike (S). membrane (M). nucleocapsid (N) and Envelope (N) proteins for positive sera grouped into 1-8 weeks post infection (wpi. upper panel) and 12-18 wpi (lower panel). IgA responses: blue; IgG responses: red; IgM responses: green. Peptides M1 - 3 are most frequent during wpi 1 - 8. peptides S286 - 289 are most frequent in wpi 12 - 18.
